# Interaction between Herpes Virus Infections and IL10 and Risk of Bone Marrow Suppression

**Published:** 2018-08-01

**Authors:** R. Yaghobi, F. Alizadeh, A. Khodavandi

**Affiliations:** 1 Shiraz Transplant Research Center, Shiraz University of Medical Sciences, Shiraz, Iran; 2 Department of Microbiology, Yasooj Branch, Islamic Azad University, Yasooj, Iran; 3 Department of Biology, Gachsaran Branch, Islamic Azad University, Gachsaran, Iran

**Keywords:** Human herpes virus 8, Cytomegalovirus, Interleukin 10, Bone marrow

## Abstract

**Background:**

Syndrome of transient bone marrow suppression may result from various extra-hematological diseases, such as immunological deregulations, and viral infectious diseases secondarily affecting the function of hematopoietic stem cells.

**Objective:**

To evaluate the pathogenic role of herpes viruses and their contraction with *IL10* cytokine gene polymorphism, which can impair hematopoiesis in patients with transient bone marrow suppression.

**Methods:**

In a cross-sectional study 30 patients who admitted to Namazi Hospital, affiliated to Shiraz University of Medical Sciences, with transient bone marrow suppression were recruited. Diagnosis of the transient bone marrow suppression was made by expert hematologists. A control group consisting of 100 healthy unrelated individuals was also included. One EDTA-treated blood sample was collected from each studied patients and plasma was isolated. The molecular prevalence of cytomegalovirus and HHV8 evaluated was evaluated using real-time and nested PCR protocols, respectively. The SNPs of the *IL10* (rs 1800896-1082G/A) cytokine gene was evaluated by PCR-RFLP method.

**Results:**

Cytomegalovirus and HHV8 infections were found in 2 and 3 of studied patients with transient bone marrow suppression. Significant higher frequency of *IL10* G allele and GG genotype were found in HHV8-infected patients comparing to uninfected ones. Higher frequencies of A allele and AG and AA genotypes of *IL10* were found in cytomegalovirus-uninfected patients comparing to infected ones, respectively. The significant higher frequencies of *IL10* AA and AG genotypes were found in controls compared to bone marrow suppressed patients.

**Conclusion:**

*IL10* genetic polymorphism might have determinative role in resistance to the cytomegalovirus, especially HHV8 infections, in patients with bone marrow suppression. Focus in new interaction between HHV8 infection and *IL10* genetics in bone marrow suppressed patients should be completed by the analysis of the anti-herpes virus immunity in future studies.

## INTRODUCTION

Syndrome of transient bone marrow suppression is a group of diseases that include a diverse group of transient clonal hematopoietic progenitor cell disorders, usually with peripheral pancytopenia [1-4]. Bone marrow suppression may result from various extra-hematological diseases such as malignancies, immunological deregulations, and infectious diseases secondarily affecting the function of hematopoietic stem cells [5]. Transient marrow suppression has been occurred in children and adults. Transient erythroblastopenia of childhood is categorized into acquired and benign [6]. Viral infections are associated with a transient reduction in the number of circulating blood cells as a consequence of bone marrow suppression. The majority of viruses inducing abnormalities of hematopoiesis are non- or poorly cytopathic for blood cells or have no known tropism for blood cell precursors. Occasional cases of hematological abnormalities caused by viruses in healthy people have also been reported [7-10]. Moreover, there is evidence suggesting a critical role of the host immune response in causing bone marrow suppression in several viral infections. Among viral infectious agents with inducing role in transient bone marrow failure, some DNA viruses may be important [11, 12]. Since earlier, the causative role of viral infections, especially human parvovirus B19, in transient bone marrow suppression and depletion of erythroid progenitor cells has been demonstrated [13]. Other viruses can participate in bone marrow suppression [12, 14-17].

Epstein-Barr virus (EBV) can be associated with bone marrow failure including single cell lineage disorders and pancytopenia mimicking acquired aplastic anemia [18-23]. EBV can also induce aplasia probably through excessive immune activation. T cells exposed to autologous EBV-infected B cells inhibit hematopoietic stem cell growth. Cytomegalovirus (CMV) associated bone marrow failure syndrome has also been rarely documented. Experimental data have shown CMV infection and replication in mesenchymal stem cells, along with an impaired stromal function [24, 25]. In spite of earlier reports, the cause of bone marrow suppression had been mostly unknown. Recent studies have also indicated the increasing evidence for immunological mechanisms, especially inflammatory cytokines against viral infections in the pathogenesis of bone marrow suppression [26-34]. Abnormal production of cytokines including interferon-γ (IFN-γ), tumor necrosis factor-α (TNF-α), and tumor growth factor-β1 (TGF-β1) causes interaction between the progenitor cells and the extra-cellular matrix of hematopoiesis, the main possible cause of immune-mediated bone marrow suppression. Interestingly, mutations occurred in the regulatory and coding regions of the cytokine genes may contribute to the hematopoietic disorders. Genetic polymorphisms in cytokine genes such as interleukin 10 (*IL10*) are also associated with resistance to some human herpesviruses. *IL10* inhibits the synthesis of the proinflammatory cytokines (such as IL1, IL6, and TNF); it also promotes antibody synthesis and cytotoxic T cell formation [35]. Therefore, in this study the pathogenic role of herpes viruses and their contraction with *IL10* cytokine gene polymorphism which can impair hematopoiesis was evaluated in patients with transient bone marrow suppression.

## MATERIALS AND METHODS

**Patients and Samples**

In this cross-sectional study, 30 patients with syndrome of transient bone marrow suppression were recruited. All of the patients were Iranians admitted to Namazi Hospital, affiliated to Shiraz University of Medical Sciences. Diagnosis of the transient bone marrow suppression was made by expert hematologists. One-hundred ethnic, sex- and age-matched healthy persons were enrolled in this study as the control group. The buffy coats and plasma were also isolated from each collected samples. Selected patients needed at least one of these indices to be included in the study: Leukopenia (WBC<3500), thrombocytopenia (platelet <150,000), and hemoglobin <14 g/dL for men and <12 g/dL for women. Reticulocyte count in bone marrow aspirate, ferritin, vitamin B_12_, and folic acid levels in blood samples were evaluated for all selected patients. Patients with other acute and chronic hematologic disorders, complications, and malignancies were excluded from this investigation. The molecular prevalence of CMV and HHV8 was evaluated using real-time and nested PCR protocols, respectively. A SNP of the *IL10* (rs 1800896-1082G/A) cytokine gene was evaluated by PCR-RFLP method. The study protocol conformed to the ethical guidelines of the 1975 Declaration of Helsinki.

**Genomic DNA Extraction**

The genomic DNA was extracted for evaluation of studied cytokine genetic polymorphisms and viruses from the collected plasma samples using DNP kit (CinnaGen, Iran) according to the manufacturer’s instruction. Beta-actin was used as internal control for evaluation of the extracted genomes. 

**Molecular Analysis of Viruses**

**CMV**

The load and diagnosis of CMV genomic DNA was done using genesig quantitative real-time PCR kit (Primer Design Ltd TM, Advanced kit, United Kingdom) by Step One Plus real-time thermocycler (Applied Biosystems-Grand Iland, NY, USA). The sensitivity of this quantitative PCR assay was enough to detect as few as 10 copy/mL of CMV genome in plasma samples. The PCR mix with total volume of 20 µL was composed of 10 µL Precision TM Master Mix (Applied Biosystems Grand I and, NY, USA), 1 µL primers and a probe targeting the glycoprotein B (gB) sequence, 1 µL primers and a probe targeting the internal control (IC) gene, 5 µL of the DNA, and 3 µL DEPS water. The thermocycling condition consisted of 1 cycle at 95 °C for 10 min, followed by 50 cycles at 95 °C for 5 sec, and 60 °C for 60 sec.

**HHV8**

The genomic DNA of HHV8 was searched in collected plasma samples using an in-house nested PCR method. Specific primer pairs were designed for amplifying a 380-bp fragment of the LANA gene. The PCR reaction mix has a total volume of 50 µL with the same ingredients in both the simple and nested PCR steps as follow: 5 µL of 10X PCR buffer, 1.5 µL MgCl_2_ (50 mM), 1 µL dNTP (10 Mm), 1 µL of each primer (20 pmol), 0.5 µL Taq (2.5 unit), and 10 µL of the sample DNA. Thermocycling program was also the same for both simple and nested PCR steps. First round at 94 °C for 5 min, 35 cycles at 94 °C for 30 sec, 55 °C for 60 sec, 68 °C for 120 sec, and final extension at 68 °C for 5 min for terminate amplification.

**Cytokine Genetic Polymorphisms**

The SNP of the *IL10* (rs 1800896-1082G/A) cytokine gene was evaluated using an in-house PCR-RFLP protocol in 25 patients with transient bone marrow suppression. The PCR mix and thermocycling conditions, and also primers used for genotyping of cytokine gene SNPs are summarized in Table 1. PCR-RFLP method in a final volume of 25 µL was employed to determine the cytokine gene SNPs. The amplified products were visualized by agarose gel electrophoresis.

**Table 1:** Primers, type of PCR, restriction enzyme and thermocycling program for the SNPs of *IL10* genotyping

Locus		Primers		Method (Restriction enzyme)	Thermocycling program	Fragment length (Base pairs)
IL10 (rs 1800896 -1082G/A)		Forward primer	5’-CTCGCTGCAACCCAACTGGC-3’	PCR-RFLP (MnlI)	95 °C, 5 min; 35 cycles 95 °C, 30 sec 60/5 °C, 45 sec. 72 °C, 1 min; 72 °C, 5 min	GG:106,33bp AA: 139 bp AG: 139, 106, 33 bp
		Reverse primer	5’-TCTTACCTATCCCTACTTCC-3’			

**Statistical Analysis**

*IL10* alleles and genotypes frequencies with herpes viral infections were calculated in patients with bone marrow suppression by direct gene counting. Statistical evaluation was also done with SPSS^®^ ver 15. Furthermore, the frequency of the alleles/genotypes was compared among the patients using χ^2^ and Fisher’s exact tests. A p value <0.05 was considered statistically significant. 

## RESULTS

Sixty percent of studied patients with bone marrow suppression were male. Studied participants had a mean age of 33 (range: 15–67) years. The control population (49% male) had a mean age of 33 years.

**Molecular Prevalence of CMV and HHV8 Infections**

The genomic DNA of CMV was found in 2 (7%) of 30 studied patients with transient bone marrow suppression. Viremia of HHV8 was found in 3 (10%) of the 30 patients. One of the patients was simultaneously co-infected with CMV and HHV8. 

**Cytokine Gene Polymorphisms and Transient Bone Marrow Suppression**

The frequency of the SNP of *IL10* (rs 1800896-1082G/A) cytokine gene was compared between males and females in patients with transient bone marrow suppression (Table 2). The frequency was also compared between patients with transient bone marrow suppression and controls (Table 3). A significantly (p=0.008) higher frequency of *IL10* (rs1800896-1082G/A) AA (low producer) genotype was found in controls compared to bone marrow suppressed patients (Table 3). The frequency of *IL10* (rs 1800896-1082G/A) AG genotypes was found to be significantly (p=0.0001) higher in controls comparing to patients (Table 3). 

**Table 2:** The frequency of the SNP of *IL-10* genotypes and alleles between male and female gender in patients with bone marrow suppression

Gene	Genotype	Female n (%)	Male n (%)	p value	OR (95% CI)
IL10 (1082)	AA	3 (30)	7 (47)	0.40	0.49 (0.06–3.50)
	GG	2 (20)	3 (20)	1.00	1.00 (0.09–10.37)
	AG	5 (50)	5 (33)	0.40	2.00 (0.29–14.38)
	A allele	11 (55)	19 (63)	0.55	0.71 (0.19–2.61)
	G allele	9 (45)	11 (37)		

**Table 3:** The frequency of *IL10* genotypes and alleles in patients with bone marrow suppression compared with controls

Gene	Genotype	Patients n (%)	Control n (%)	p value	OR (95% CI)
IL10 (1082)	AA	10 (40)	16 (16)	0.008	3.50 (1.20–10.19)
	GG	5 (20)	5 (5)	0.01	4.75 1.06–21.45)
	AG	10 (40)	79 (79)	<0.001	0.18 (0.06–0.49)
	A allele	30 (60)	111 (55.5)	0.56	1.20 (0.61–2.37)
	G allele	20 (40)	89 (44.5)		

**HHV8 Infection and Cytokine Gene Polymorphisms**

The frequency of the SNP of the *IL10* (rs 1800896-1082G/A) cytokine gene was compared between HHV8 infected patients *vs*. uninfected patients with bone marrow suppression (Table 4). A significant higher frequency of *IL10* (rs1800896-1082G/A) G allele (p<0.001) and GG (high producer) (p=0.001) genotype was found in HHV8-infected patients comparing to uninfected ones (Table 4). 

**Table 4:** The frequency of the SNP of *IL10* genotypes and alleles between HHV8 infected and uninfected patients with bone marrow suppression

Gene	Genotype	HHV8^+^ n (%)	HHV8^–^ n (%)	p value	OR (95% CI)
IL10 (1082)	AA	0 (0)	9 (43)	0.15	0.00 (0.00–3.98)
	GG	3 (100)	2 (10)	<0.001	—
	AG	0 (0)	10 (48)	0.11	0.00 (0.00–3.26)
	A allele	0 (0)	28 (67)	0.001	0.00 (0.00–0.55)
	G allele	6 (100)	14 (33)		

**CMV Infection and Cytokine Gene Polymorphisms**

The frequency of the SNP of the *IL10* (rs 1800896-1082G/A) cytokine gene was compared between CMV infected and uninfected patients with bone marrow suppression (Table 5). Higher frequencies of *IL10* (rs1800896-1082G/A) A allele and AG and AA genotypes were found in CMV-uninfected patients comparing to infected ones (Table 5).

**Table 5:** The frequency of the SNP of *IL10* genotypes and alleles between CMV infected and uninfected patients with bone marrow suppression

Gene	Genotype	CMV^+^ n (%)	CMV^–^ n (%)	p value	OR (95% CI)
IL10 (1082)	AA	1 (50)	9 (38)	0.72	1.67 (0.00–71.62)
	GG	0 (0)	5 (21)	0.47	0.00 (0.00–22.23)
	AG	1 (50)	10 (42)	0.81	1.40 (0.00–59.74)
	A allele	3 (75)	28 (58)	0.51	2.14 (0.17–57.69)
	G allele	1 (25)	20 (42)		

## DISCUSSION

Interaction between viruses and antiviral host immune responses plays as an introducing and complicating role in transient reduction of circulating blood cells and bone marrow suppression. However, controversies exist about the importance of antiviral immunity and pathogenesis of bone marrow suppression, especially with focus on genetics of inflammatory cytokines against viral infections [14, 15, 26-32]. Cytokines including IFN-γ, TNF-α, TGF-β1, and *IL10* directly and indirectly induce impairment in progenitor cells and the extracellular matrix of hematopoiesis and immune-mediated bone marrow suppression. Infection of viruses can participate in bone marrow suppression [12, 14-17]. Rearrangement in the regulatory and coding regions of cytokine genes may also contribute to the hematopoietic disorders. In contrast, based on earlier reports, *IL10* genetic polymorphisms may associate with resistance to some viral infections. *IL10* inhibits the synthesis of the proinflammatory cytokines and promotes antibody synthesis and cytotoxic T cell formation [33]. Therefore, the importance of genetic reprograming in cytokine genes in interaction with herpes viral infection was focused in patients with bone marrow suppression. 

In this study, similar pattern of *IL10* (rs1800896-1082G/A) genetic polymorphisms were found in bone marrow suppressed patients resistant to HHV8 and CMV infections. Significant higher frequency of the A allele and AA and AG (low producer) genotypes of *IL10* (rs1800896-1082G/A) were found in patients resistant to HHV8 infection compared to infected ones (Table 4). The AA and AG (low producer) genotypes were also found with higher frequencies in studied patients resistant to CMV infection. These homologous (AA) and heterologous (AG) low producer genotypes of *IL10* (rs1800896-1082G/A) also presented with a significantly higher frequency in normal controls who not experienced CMV and HHV8 infections compared to bone marrow suppressed patients (Table 3). Similar to this report, in other studies, genetics polymorphisms in *IL10* gene are associated with resistance to some human herpes viruses [33]. The promoter of *IL10* gene contains SNPs including G/A at -1082, C/T at -819, and C/A at -592, with formation of GCC, ACC, and ATA haplotypes. The frequency of the ACC haplotype increased resistance to CMV infected patients. But in persons without EBV infection GCC haplotype was found with higher frequency. Interestingly, the ATA haplotype seemed to have an increased resistance to herpes simplex virus (HSV) infection [35]. *IL10* genetics has a role in the resistance to EBV in early childhood [34]. In a recent study, the AA (low producer) genotype of *IL10* (rs 1800896-1082G/A) polymorphism was found with higher frequency (representing the ATA and ACC haplotypes) in patients hospitalized for severe EBV infection compared to normal controls [35]. In another study, the ATA (low producer) haplotype were more frequent in patients infected with (Varicella zoster virus, VZV) [36]. The increased level of *IL10* is responsible for the severity of EBV and VZV infections [33].

In conclusion, *IL10* genetics had a significant role in resistance to the herpes virus infections in patients with bone marrow suppression. Results suggested that similar defense mechanisms operate in the primary resistance against CMV and HHV8 infections. Moreover, early protection against CMV and HHV8 infections and complete resistance might regulate the same immunopathogenic pathways. Therefore, for new analysis of the interaction between HHV8 infection and *IL10* genetics in bone marrow suppressed patients, these observations should be completed by the analysis of the herpes virus immunology related antiviral mechanisms in future studies.
